# Awareness, Perceptions, Gaps, and Uptake of Maternity Protection among Formally Employed Women in Vietnam

**DOI:** 10.3390/ijerph19084772

**Published:** 2022-04-14

**Authors:** Tuan T. Nguyen, Jennifer Cashin, Ha T. T. Tran, Duong H. Vu, Arijit Nandi, Minh T. Phan, Nguyen D. C. Van, Amy Weissman, Toan N. Pham, Binh V. Nguyen, Roger Mathisen

**Affiliations:** 1 Alive & Thrive Southeast Asia, FHI 360, Hanoi 11022, Vietnam; jcashin@fhi360.org (J.C.); vduong@fhi360.org (D.H.V.); aweissman@fhi360.org (A.W.); rmathisen@fhi360.org (R.M.); 2 Research and Training Center for Community Development, Hanoi 11616, Vietnam; ha.tran@rtccd.org.vn (H.T.T.T.); van.nguyen@rtccd.org.vn (N.D.C.V.); 3 Department of Epidemiology, Biostatistics and Occupational Health and Institute for Health and Social Policy, McGill University, Montreal, QC H3A 1A3, Canada; arijit.nandi@mcgill.ca; 4 Department of Legal Affairs, Ministry of Labor, Invalids and Social Affairs (MOLISA), Hanoi 11022, Vietnam; phanthanhminh_l@yahoo.com (M.T.P.); vanbinhir@gmail.com (B.V.N.); 5 Asia Pacific Regional Office, FHI 360, Bangkok 10330, Thailand; 6 Institute of labor Science & Social Affairs, Ministry of Labor, Invalids and Social Affairs (MOLISA), Hanoi 11022, Vietnam; ngoctoan.tkt@gmail.com

**Keywords:** breastfeeding, gender equality, maternal and child health, maternity protection, public policy, parental leave, Vietnam

## Abstract

Maternity protection is a normative fundamental human right that enables women to combine their productive and reproductive roles, including breastfeeding. The aim of this study is to examine the uptake of Vietnam’s maternity protection policy in terms of entitlements and awareness, perceptions, and gaps in implementation through the lens of formally employed women. In this mixed methods study, we interviewed 494 formally employed female workers, among whom 107 were pregnant and 387 were mothers of infants and conducted in-depth interviews with a subset of these women (*n* = 39). Of the 494 women interviewed, 268 (54.3%) were working in blue-collar jobs and more than 90% were contributing to the public social insurance fund. Among the 387 mothers on paid maternity leave, 51 (13.2%) did not receive cash entitlements during their leave. Among the 182 mothers with infants aged 6–11 months, 30 (16.5%) returned to work before accruing 180 days of maternity leave. Of 121 women who had returned to work, 26 (21.5%) did not receive a one-hour paid break every day to express breastmilk, relax, or breastfeed, and 46 (38.0%) worked the same or more hours per day than before maternity leave. Although most women perceived maternity leave as beneficial for the child’s health (92.5%), mother’s health (91.5%), family (86.2%), and society (90.7%), fewer women perceived it as beneficial for their income (59.5%), career (46.4%), and employers (30.4%). Not all formally employed women were aware of their maternity protection rights: women were more likely to mention the six-month paid maternity leave (78.7%) and one-hour nursing break (62.3%) than the other nine entitlements (2.0–35.0%). In-depth interviews with pregnant women and mothers of infants supported findings from the quantitative survey. In conclusion, although Vietnam’s maternity protection policy helps protect the rights of women and children, our study identified implementation gaps that limit its effectiveness. To ensure that all women and their families can fully benefit from maternity protection, there is a need to increase awareness of the full set of maternity entitlements, strengthen enforcement of existing policies, and expand entitlements to the informal sector.

## 1. Introduction

Maternity protection, a fundamental human right that enables women to combine their reproductive and productive roles without prejudice or damage to their economic and social security, is normative globally [1,2]. The International Labor Organization (ILO)’s Maternity Protection Convention, 2000 (No. 183) and its accompanying Recommendation (No. 191) call for the establishment of an integrated set of essential maternity protection measures, including: essential health services for pregnant women, mothers, and children; paid maternity leave with cash support equal to or greater than two-thirds previous earnings, financed through compulsory social insurance or public funds; protection from discrimination at work and from working conditions that may be harmful to mother or child; breastfeeding breaks and facilities; and the right to return to the same paid position at the same salary rate [1,2]. While nearly every country in the world has adopted some form of maternity protection legislation, only about half of all countries meet the minimum 14 weeks (Convention 183), and less than a quarter meet the recommended 18 weeks or more (Convention No. 191) [1]. Sources of funding for maternity entitlements also vary: from public financing, employers, employee contributions, and mixed [1]. Some policies provide support to women after returning to work, while others do not [1].

Maternity leave positively impacts maternal mental and physical well-being [3,4]. Paid parental leave, including paternity leave, is associated with a more gender-equitable division of domestic labor and increased paternal involvement in child rearing [5], as well as increased earnings by mothers [6]. Research shows that equitable parental leave could help to close the ‘wage gap’ between men and women by helping mothers return to the same position after childbirth [7]. The adoption of family-friendly policies that allow for a more equitable and efficient distribution of time among family members will attract more women to the workforce by reducing constraints on women’s time and child health [8] and positively impact economies through increased female labor force participation and productivity [9,10].

Increasing the duration of paid maternity leave is associated with a higher prevalence of early initiation of breastfeeding, exclusive breastfeeding among infants under six months, and a longer duration of breastfeeding [11], as well as reduced neonatal, infant, and child mortality rates in low, middle, and high-income countries [12,13,14]. Conversely, short maternity leave (less than six weeks or 6–12 weeks) is associated with failure to initiate breastfeeding and early cessation of breastfeeding [15]. Multiple studies have shown that providing working mothers with time, space, and support for breastfeeding when they return to work can increase breastfeeding duration and adherence to recommended breastfeeding practices [16,17,18].

Vietnam is a lower-middle-income country in Southeast Asia with a population of more than 95 million and a female labor force participation rate (70.9% as of 2019) that is significantly higher than the global (47.2%) and Asia Pacific regional average (43.9%) [19]. Vietnam has advanced policies on gender equality [20] and maternity protection compared with other countries in the world [1], having recognized the right of working women to paid maternity leave since the country’s establishment in 1945 [21,22]. The duration of paid maternity leave in Vietnam has increased over time, from 42 days (1947–1960), to 60 days (1961–1984), and 180 days (1985–1994) [21,23,24,25]. Due to pressure from the private sector, the duration of maternity leave decreased to 120 days from 1994 to 2012 [26] before reverting to 180 days from 2013 onwards [27,28,29]. Women in Vietnam who contribute to the public social insurance fund for at least six months in the previous 12 months prior to birth are eligible for six months (180 days) paid maternity leave funded through social insurance in addition to other entitlements, including: paid breastfeeding breaks, job security, and five standard free prenatal check-ups [27,28,29]. After 120 days of maternity leave, women have the option to return to work early with a doctor’s approval and in agreement with their employers [27,29]. Effective in 2016, the Law on Social Insurance was updated to include up to 50 fully paid days off for abortion, miscarriage, stillbirth, or complicated pregnancy, and up to 15 fully paid days off for family planning [28]. According to the law, fathers who contribute to the social insurance fund are eligible for five days of paid leave if their partners have a vaginal birth; seven days of paid leave for cesarean or preterm birth; 10 days of paid leave for twins and an additional three days per child for higher order multiples; or 14 days for the cesarean birth of twins, all with full pay [28]. The 2019 revised Labor Code maintains these regulations and clearly prohibits discrimination based on pregnancy, marital status, family responsibilities, and other employee characteristics [29].

To benefit from maternity protection policies, female workers must be aware of their rights and feel entitled and encouraged to utilize them [30]. Although paid maternity leave and other entitlements are statutory in Vietnam, evidence from other contexts indicates that women’s perceptions of the accessibility and feasibility of maternity entitlements are important determinants of uptake [31,32]. In a recent study in Korea, a supportive workplace culture, higher pay, and membership in a labor union were significant determinants of the perceived accessibility of maternity leave [31].

Since Vietnam’s amended maternity leave policy took effect on 1 January 2013, data on its implementation and uptake have been lacking in peer-reviewed literature. While a recent study explored perceptions of national stakeholders on policies to protect breastfeeding, including the expanded paid maternity leave policy [33], there has been no research to explore female workers’ perceptions of the policy [33]. To address this gap, we conducted this study to examine the uptake of Vietnam’s maternity protection policy in terms of entitlements and awareness, perceptions, and gaps in implementation through the lens of formally employed women.

## 2. Methods

### 2.1. Study Design

This mixed methods, cross-sectional study employed a quantitative survey and in-depth interviews with pregnant women and mothers of infants 0–11 months of age [34].

### 2.2. Setting

Researchers purposely selected two provinces and one municipality to capture various levels of socio-economic development in Vietnam. Bac Ninh, in the Red River Delta Region, is transforming from an agricultural to a more industrialized province in the north. Binh Duong, in the Southeastern Region, is an industrial province in the south, and Ho Chi Minh City (HCMC) is the most populous city in Vietnam (south). The population of Bac Ninh is about 380,000, with 28% living in urban areas, while the population of Binh Duong is 2,460,000 with about 80% living in urban areas. HCMC has a population of 9,040,000, with 80% living in urban areas [35].

### 2.3. Participants and Sample Size

A detailed description of the study participants and sampling procedure has already been published [34,36]. In summary, for the quantitative survey, we employed a three-stage cluster sampling design in each of the selected provinces. Stage 1—Selection of districts: we selected three districts in each province using simple random sampling. Stage 2—Selection of clusters: we defined a sample frame of clusters and used probability proportional to size cluster sampling to select 30 clusters in the selected districts. Stage 3—Selection of participants: the research team identified pregnant women and mothers of infants aged 0–5 and 6–11 months in each cluster using systematic random sampling [34]. We did not have any exclusion criteria. Researchers used tablets to interview 994 women using structured questionnaires. The overall response rate was 85.4%. Data from a subset of 494 women working in the formal sector (in blue- or white-collar jobs, which typically require labor contracts), including 107 pregnant women and 387 mothers of infants, were used in these analyses. The research team also conducted in-depth interviews with a purposively selected subset of 39 women, 9 of whom were pregnant and 30 of whom had infants, who had also participated in the quantitative survey. The interviews were audio recorded with the consent of participants. To ensure data quality, privacy, and confidentiality, all interviews were conducted in private spaces with only respondents and interviewers present, outside respondents’ workplaces.

Each quantitative or in-depth interview took about 45 min to complete. At the end of each interview, we gave VND 100,000 (equal to USD 4.5) to compensate for their travel expenses and time. The interviewees were offered a gift, such as a parenting book or a raincoat [34,36].

To ensure data quality and compliance with the study research protocol, two supervisors and enumerators were trained and supervised by investigators from Alive & Thrive (A&T) and the Research and Training Centre for Community Development (RTCCD) [34,36].

### 2.4. Variables and Themes

For the quantitative survey, we adapted standardized questionnaires based on the ILO Maternity Protection Resource Package, Module 13: Assessing Maternity Protection in Practice [1] and regulations included in the Vietnam Labor Code 2012 [27]. The questionnaires had been pretested by RTCCD in the field outside the study locations. A complete research protocol including questionnaires has already been published in a peer-reviewed journal [34].

To evaluate the uptake of the maternity protection, we asked about respondents’ experiences related to maternity protection policies during pregnancy, at birth, during maternity leave, and after returning to work. To evaluate awareness of the rights of working women, we used a multiple-choice question (i.e., “Can you tell me the rights of a working woman who is pregnant or breastfeeding an infant under 12 months as indicated in the Vietnam Labor Code?”) without reading any responses. Interviewers recorded all appropriate responses. To evaluate women’s perceptions of the policy, we used a Likert-scale question on the profitability (1: very unprofitable to 6: very profitable) of each policy for diverse groups and outcomes. For example: “In your opinion, what is the profitability of the policy (i.e., one hour breastfeeding break during the first year after child’s birth) on the following outcomes?”: (1) overall benefit for society, (2) employers, (3) mother’s income, (4) child health, (5) mother’s health, (6) mother’s career, (7) mother’s preference on connecting with friends and coworkers, and (8) the whole family [34].

We collected characteristics of study participators, such as age, ethnicity, marital status, education, and employment status (farmer, blue-collar, white-collar, small trader/self-employed, unemployed/housemaker/student or other).

For in-depth interviews with women, we further discussed: (1) perceptions and experiences of breastfeeding and maternity protection policies during pregnancy, at birth, in the first six months, and after returning to work, (2) suggestions for improvement, and 3) sharing responsibilities related to childcare and domestic tasks.

### 2.5. Data Management and Analysis

We de-identified data prior to analysis. Descriptive data analyses with stratification by job type (e.g., blue- vs. white-collar) and by stages related to maternity protection (e.g., pregnant women, mothers of infants aged 0–5 months or 6–11 months) were conducted using Stata, Version 15.0 (Stata Corp LP, College Station, TX, USA). All qualitative interviews were recorded. Records were transcribed fully by an independent team. A deductive thematic approach was used to categorize findings into themes (e.g., awareness, perceptions, and uptake of entitlements) using NVivo 11. Illustrative quotes were drawn from thematic sheets.

## 3. Results

### 3.1. Over 90% of Formally Employed Women Contribute to the Public Social Insurance Fund

In total, 494 formally employed women were interviewed during the quantitative survey: 107 pregnant women and 387 mothers with infants. Most participants were ethnic majority Kinh (around 92%), married (nearly 100%), and living with their husbands or partners (around 95%). One third of women completed a bachelor’s degree or higher. More than one-half of pregnant women sampled (54.2%) and 43.4% of mothers were working in white-collar occupations. Over 90% of respondents reported contributing to the public social insurance fund (90.7% among women with blue-collar jobs and 92.9% among women with white-collar jobs) (Table 1).

### 3.2. Not All Formally Employed Women Are Aware of Their Maternity Protection Rights

**Figure 1:**
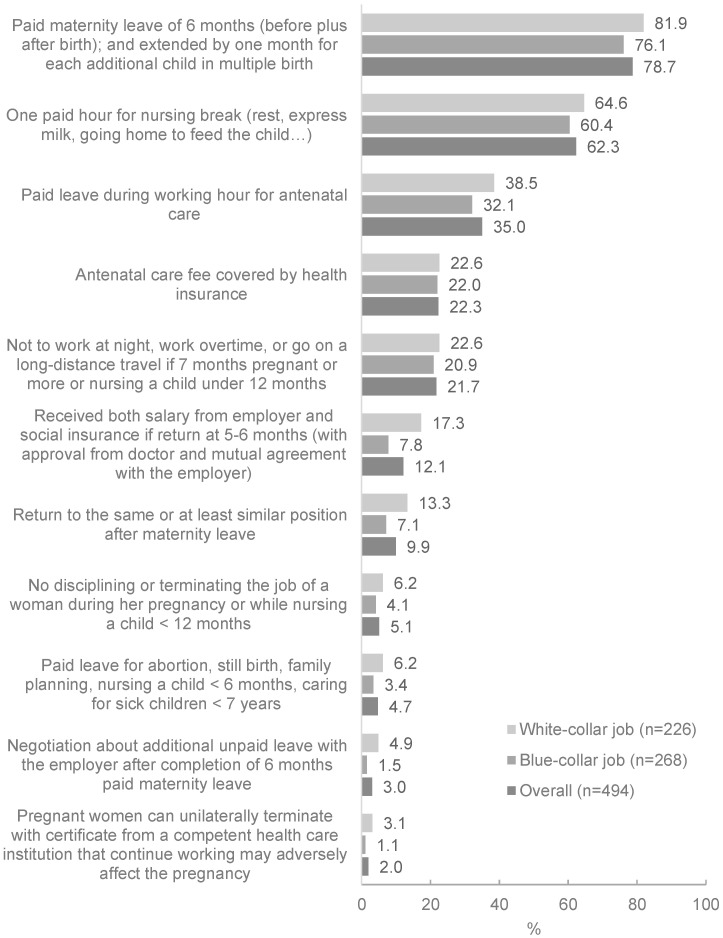
Awareness about maternity protection rights of working women, among pregnant women, and mothers of infants 0–11 months of age, by job category.

Four out of five pregnant women and mothers surveyed were aware of their right to paid maternity leave for six months (Figure 1).

In-depth interview respondents who were working in large companies or enterprises in industrial zones reported that their companies supported them to utilize their maternity entitlements as provided by law. According to women working in such companies, human resource or accounting departments are typically responsible for verbally informing female employees of their entitlements, while some companies provide written documents detailing these entitlements to female employees.

“Women are allowed to take five days off for pregnancy care, six months maternity leave, and one hour off per day for breastfeeding from the standard working hours. This information I learned from websites. When I was due, my husband had five days off with full salary paid as well.”

Worker and mother of a 6–11-month-old infant, Binh Duong.

However, the situation is different in some companies, where women were not always informed of their entitlements and relied on peers for information, leaving them at risk of not receiving their full entitlements.

“I am working for a [foreign-name omitted]-owned enterprise, and provided six months maternity leave, five days off for prenatal care with full payment… This company has several hundred female workers, there is a trade union, but the trade union [representative] did not inform us about [maternity] benefits [for workers].”

Worker and mother of a 0–5-month-old infant, Binh Duong.

“… I explored the information from the internet and peers in the unit. The human resource division did not inform me of the maternity benefits until I came to ask about them, then they provided detailed information.”

Worker and mother of a 6–11-month-old infant, Bac Ninh.

Two thirds of women surveyed were aware of their right to a one-hour breastfeeding break after returning to work, until their child reached one year of age (Figure 1). Fewer women were aware of their rights to paid leave during working hours for routine antenatal care (35.0%); antenatal care fees being covered by health insurance (22.3%); not working at night or overtime or traveling long distances if seven or more months pregnant (21.7%); receiving both salary from their employer and social insurance if they return to work before six months (12.1%); and returning to the same or similar position after maternity leave (9.9%). Few quantitative survey respondents were aware of their rights to: paid leave for abortion, still birth, or family planning (4.7%); negotiate for additional unpaid leave after completion of six months maternity leave (3.0%); and unilaterally terminate or temporarily suspense of their employment contract (2.0%) (Figure 1). Awareness of maternity entitlements tended to be higher among women with white-collar jobs (Figure 1).

Some respondents perceived that maternity protection entitlements provided by law and financed through their contributions to the public social insurance fund were benefits provided by their employers.

“In addition to government-issued benefits, the company has other good benefits for pregnant workers: no overtime work, no queuing in any event (the company has priority lane for pregnant women), giving pregnant woman a position that is flexible for sitting, and priority for early lunch.”

Worker and mother of a 6–11-month-old infant, Bac Ninh.

### 3.3. Not All Mothers on Paid Maternity Leave Receive Cash Entitlements When They Need Them Most

**Figure 2:**
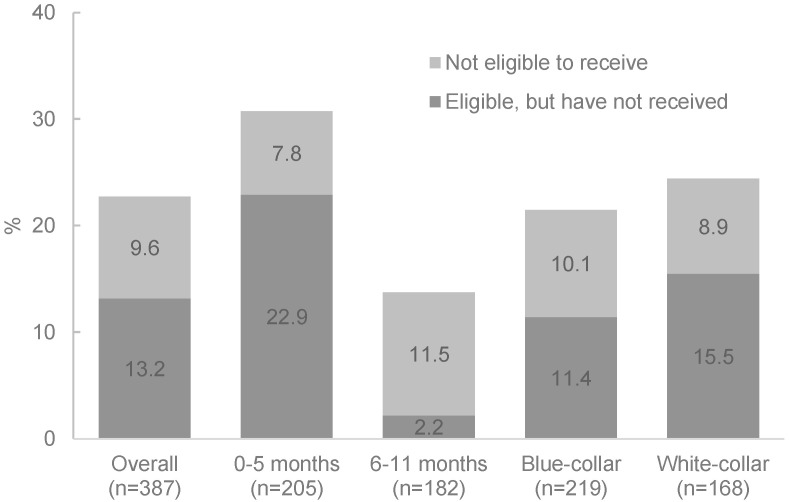
Percentage of women in formal sector who have not received cash support after childbirth, by job category, and infant age.

Although most survey respondents took maternity leave (nearly 99%), only 69.3% of women with an infant aged 0–5 months and 86.3% of women with an infant aged 6–11 months had received cash entitlements during their maternity leave (Figure 2). Almost a quarter (22.9%) of mothers with an infant 0–5 months of age who were eligible to receive cash through their contribution to the public social insurance fund had not yet received it (Figure 2).

Among mothers of infants aged 6–11 months who received cash entitlements and had already returned to work (*n* = 101), 98.0% had received cash payments for at least six months. For 82.2% of these mothers, cash entitlements were equal to or greater than two-thirds their previous earnings, as recommended by ILO. Nevertheless, 12.6% of mothers (*n* = 387) perceived that having a child negatively affected their family’s ability to pay for necessary expenses, with a similar prevalence for blue- and white-collar workers (Data not shown).

Most in-depth-interview participants reported that cash entitlements were wire transferred to their bank accounts during maternity leave. For those without bank accounts, the money was sent to them from the social insurance office via their employers either during maternity leave, when the woman went to the office to collect cash from the accountant, or when she came back to work after six months.

“I was allowed a day-off to get antenatal care check-ups. When I showed the doctor’s paper, the day-off was counted for salary payment. After delivery, I was transferred the cash benefits and allowed six months to take care of the baby.”

Worker and mother of a 6–11-month-old infant, HCMC.

However, one mother who did not receive cash entitlements during her maternity leave observed incorrect practices by her employer and potential misuse of social insurance funds.

“Previously the maternity cash benefits were paid twice a year for all post-partum workers, at the middle and end of the year. However, the cash benefit is now paid once per year, at the end. We guess that the company uses our money to turn over for business. That is our gossip. They get money from the insurance agency but they keep it; what can we do? Six months at home without money [is really a challenge of life].”

Worker and mother of a 0–5-month-old infant, Bac Ninh.

### 3.4. Some Mothers Return to Work before Six Months after Birth

We found that 4.9% (10/205) of mothers with infants aged 0–5 months (8.1% and 2.5% among white- and blue-collar workers, respectively) had already returned to work. Among mothers with infants aged 6–11 months, 16.5% (30/182) had returned to work before accruing 180 days of maternity leave (22.0% and 12.0% among white- and blue-collar workers, respectively).

Mothers who returned to work early were more likely to report a health condition related to insufficient recovery from birth within the first month of returning to work: 36.7% of mothers with infants 6–11 months of age who returned to work early (*n* = 30) reported such a health condition, compared with 30.6% of those who took the full 180 days or longer of maternity leave (*n* = 152; Data not shown). Of 121 women who had returned to work, 26 (21.5%) did not receive a one-hour paid break each day to express breastmilk, relax, or breastfeed and 46 (38.0%) worked the same or more hours each day than before maternity leave. Qualitative interviews also confirmed this finding.

**Figure 3:**
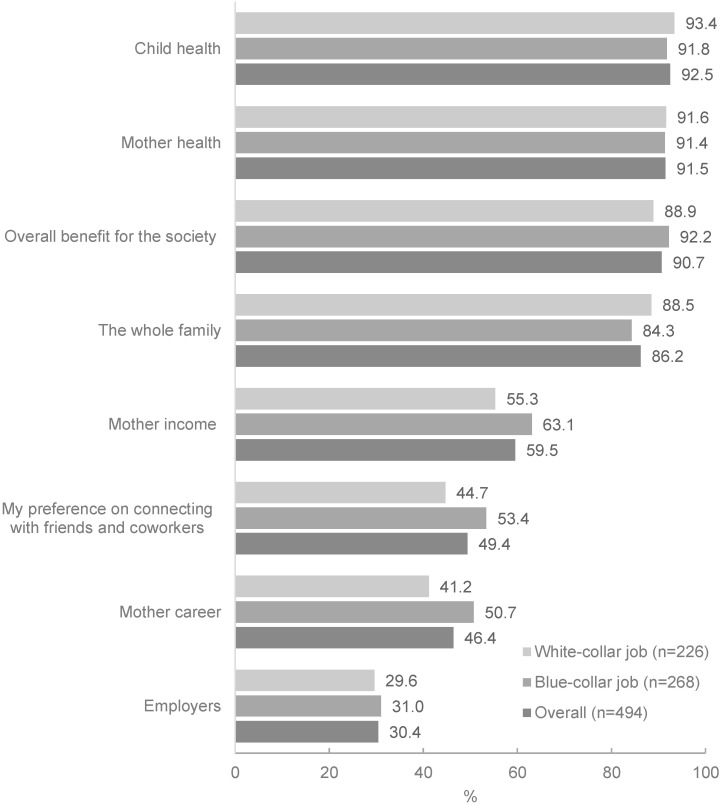
Proportion of formally employed women who perceived maternity leave policies profitable or very profitable with respect to themselves, their families, their employers, and society.

While most respondents strongly agreed that maternity leave policies were beneficial to maternal health (91.5%) and child health (92.5%), society (90.7%), and the family (86.2%) (Figure 3), fewer women felt that the policy was beneficial for: their income (59.5%), connecting with friends and co-workers (49.4%), their career (46.4%), and their employers (30.4%) (Figure 3). Women working in white-collar jobs were less likely to perceive maternity leave as being beneficial for their income, connecting with colleagues, and advancing their career than those working in blue-collar jobs.

In line with these findings, qualitative interviews revealed that some women want to return to work before taking the full 180 days of paid leave to earn more income for their families or because they had not received cash, as mentioned above. However, very few were able to return to work at their companies prior to reaching 180 days of maternity leave, and as a result, some women reported seeking informal jobs during their maternity leave.

“I returned to work early [before child six months of age] due to insufficient income. My husband is a worker too, so the income is not sufficient for the family. Therefore [we] registered for extra hourly work to increase the income.”

Worker and mother of a 0–5-month-old infant, Binh Duong.

### 3.5. Discriminatory Hiring and Employment Practices Are Reported

From the perspective of employed women, pregnancy, childbirth, and child rearing are barriers to employment.

“The [firm—name omitted] often prefer unmarried staff or staff without child-related issues so that the work will not be affected. If on leave, they will need only one or two days off. For maternity leave, it is long. Upon returning to work, [the staff] comes late and goes home early or have distractions.”

Mother of a 0–5-month-old infant, with a white-collar job, HCMC.

**Figure 4:**
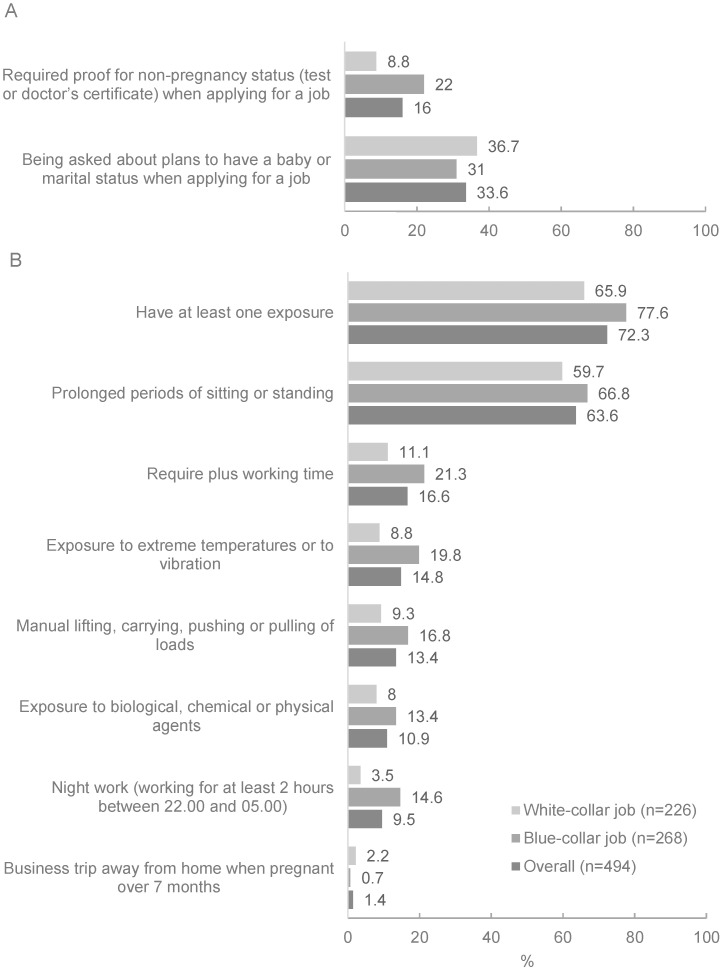
Proportion of formally employed women who reported an unfavorable situation during their job application (**A**) and at work (**B**).

During the job application process, 16.0% of survey respondents were asked to confirm that they were not pregnant and 33.6% of them were asked about their marital status or plans to become pregnant (Figure 4).

This finding was reinforced by qualitative interviews, in which women shared their experiences of being asked about their plans related to pregnancy or being requested not to become pregnant during their first two years with the company during their job interview. These questions were typically asked in a polite and friendly way and women tended to respond without any doubt or refusal, not being aware of their right to refuse to answer.

“The company just required the health-check up certificate, not a pregnancy test. However the company requested me not to get pregnant during the first two years of working.”

Worker and mother of a 0–5-month-old infant, Bac Ninh.

Even after the hiring process, some companies have specific articles in contracts or employment agreements relating to marriage, pregnancy, birth, or childbearing, which can be a barrier for women to maintain their position.

“In the contract with the [firm—name omitted] there is an article that labourer is not allowed to get pregnant in the first two years. If I have a baby it means I break the contract agreement. There will be two options. Option 1, the contract will be terminated. Option 2, I will talk with the division head to support me for six months of maternity leave to ask other staff to cover my role.”

Mother of a 0–5-month-old infant, with a white-collar job, HCMC.

Qualitative interviews revealed that some companies circumvent the law against dismissing pregnant women and women with infants under one year of age by convincing women to spontaneously quit their jobs.

“I knew the basic maternity benefits such as six months maternity leave, back to previous position after maternity leave, no termination of contract in the first one year after birth. However, the [firm—name omitted] politely required me to write a letter of resignation and sign it. When all the paperwork is done, they would return the social insurance book.”

Mother of a 0–5-month-old infant, with a white-collar job, HCMC.

### 3.6. Some Women Report Working Conditions That Are Inappropriate for Pregnant Women and Mothers of Infants

Three quarters of respondents experienced at least one unfavorable condition at work, mostly prolonged periods of sitting or standing. The percentage of women who were exposed to manual lifting, carrying, pushing, or pulling of loads, exposure to biological, chemical, or physical agents, exposure to extreme temperatures or vibration, and night work ranged from 8% to 18% (Figure 4). Less than 3% of participants experienced other hazards or unhealthy conditions and business trips away from home (Figure 4). Women working in blue-collar jobs were more likely to report unfavorable conditions at work (Figure 4).

**Figure 5:**
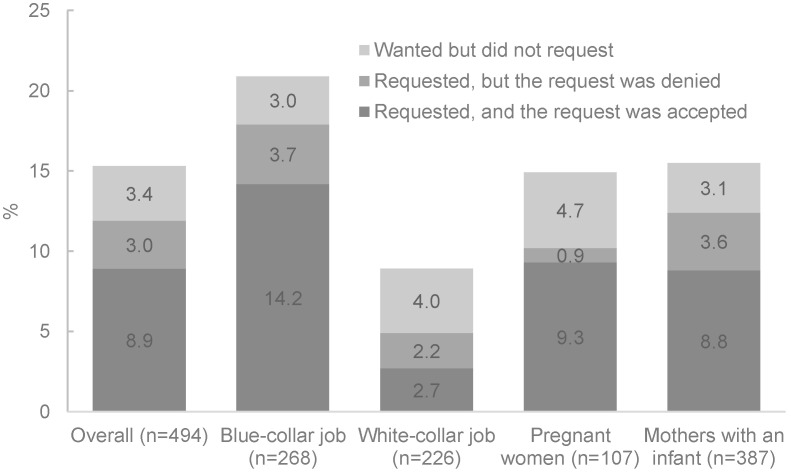
Request a more appropriate duty or moving to a safer job and its results.

Among survey respondents, 85% did not request a lighter duty because they found that their current job was safe and suitable (Figure 5). The prevalence tended to be higher in women with a white-collar job (91.2%) than those with a blue-collar job (79.1%). Among those who requested a change in their duties, requests were more commonly granted for blue-collar workers (14.2%) than white-collar workers (2.7%). However, across job categories, 6.4% of respondents did not request a change in their duties, despite wanting to, or made a request that was denied (Figure 5).

**Figure 6:**
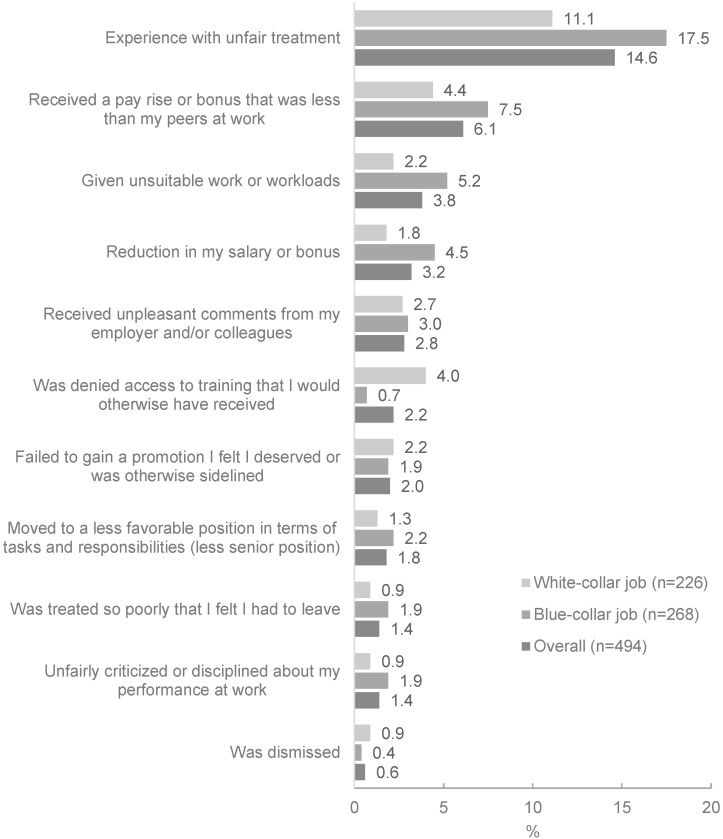
Perceived unfair treatment at work among formally employed pregnant women and mother with infants by job category.

Almost 15% of respondents experienced at least one situation of unfair treatment at work, and the prevalence was higher among women in blue-collar jobs (17.5%) than in white-collar jobs (11.1%). The most common forms of unfair treatment reported were unsuitable workloads, less favorable positions, lower salary or bonus, and unpleasant comments from employers or colleagues (Figure 6).

“In my company, we are allowed for 10 min to go to rest room every two hours. This regulation applies for both pregnant women and non-pregnant persons. In my unit, I must stand still for a long time, so my pregnancy was a problem. Other pregnant women in my company were allowed to sit more during pregnancy but I was not… We neither provided feedback nor asked [the company human resource] as the director would assign [the issue] to the supervisor [to address]. The supervisor would change their attitude [toward us], and we are under the pressure of the supervisor.”

Worker and mother of a 0–5-month-old infant, Binh Duong.

## 4. Discussion

Results from our study showed that the awareness and uptake of maternity protection entitlements is high among formally employed women in Vietnam and that most women perceive maternity protection policies as beneficial. Most women in our sample benefited from the full 180 days of maternity leave and returned to the same positions and pay they held prior to childbirth. However, we also identified implementation gaps that potentially reduce the effectiveness of these policies and negatively impact working women’s experiences. Here, we analyze the findings and experiences of respondents during various stages, from applying for a job to taking maternity leave and returning to work, within the broader context of Vietnam’s labor market and national efforts to promote gender equality and improve maternal and child health.

During pregnancy, most women in our sample reported that they found their jobs suitable during pregnancy, or if the work was unsuitable, their request to change was granted. However, not all women are aware of their full entitlements—especially those related to their right to return to the same position and pay after maternity leave, to use paid leave for family planning, and to negotiate for additional unpaid leave—meaning that they are at risk of not receiving these entitlements. Women in our sample who work in small companies reported reliance on peers for information about maternity entitlements, again leaving them at risk of not receiving their protections accounted for by law. One in-depth interview respondent reported disciplinary action leading to job termination due to pregnancy, childbirth, or maternity leave, in violation of the Vietnam Labor Code which prohibits disciplining or firing a female employee during their pregnancy and in the first 12 months after her birth [27,29]. Similar unethical practices were recently reported in China, where female employees were disciplined for the ‘untrustworthy’ behavior of becoming pregnant without informing their employers in advance [37]. Employers who violate the Labor Code are required to pay a fine of 10–20 million VND (or 440–880 USD) and reinstate employees in their previous posts [38]. Our findings call for the need to communicate to workers more effectively about their rights and strengthen the monitoring and enforcement of violations.

While on maternity leave, most women in our sample reported receiving cash entitlements equal to or greater than two thirds their previous earnings, in line with ILO recommendations [1,2,39]. However, a considerable proportion of women do not receive their cash support when they need it most, during their leave from work. This could be due to several factors, including: the employer submitting the entitlement package to the social insurance office late; the employee lacking a bank account to receive funds or being unable to access her bank accounts from her hometowns while on leave; or the employee submitting evidence of childbirth late. Our findings call for a more streamlined digital system to submit documents and facilitate the transfer of funds online to women who are on maternity leave. In some cases, women were not eligible to receive cash support because their employers did not contribute or contributed late to the public social insurance fund, suggesting that there is a need for strengthened monitoring and enforcement to identify and penalize employers that do not comply with local laws and regulations. In other cases, female employees were ineligible for cash support because they had not contributed to (i.e., they work in the informal sector) or contributed for less than 6 months to the public social insurance fund [27,28,29]. With an estimated two thirds of the labor force in Vietnam ineligible for paid maternity leave [19], many families are unable to benefit from paid parental leave [27,28,29]. Extending maternity protection and support to female workers in the informal sector, those who have not contributed to the social insurance fund long enough to receive entitlements, or to all women regardless of employment status, could improve breastfeeding rates and related health outcomes, while making vulnerable households more resilient to financial shocks and contributing toward social and health equity [33,39,40,41].

Some women in our sample, especially those working in white collar jobs, perceived that taking six months maternity leave as not accessible or feasible to them. These women reported needing to find additional work during their maternity leave or returning to work early because their pay during maternity leave was not enough to meet their family’s needs. This is because maternity cash entitlements are based on monthly income contributed to the public social insurance fund prior to childbirth, not an employee’s total income that typically includes bonuses or overtime allowances [27,28,29]. Although it is not required by law, we also found that some employers provide additional cash support to employees during maternity leave (12% received additional money from the employers), which may help to reduce the number of women returning to work before they have taken their full 180 days of entitled leave.

Some women feel that six months maternity leave is not feasible for them due to non-financial reasons. Findings from our study showed that women who perceive maternity leave to be detrimental to their careers (more than half of the women) and employers (more than two thirds of the women) are more likely to return to work early. About half of the women in our sample preferred to return to work before completion of six months maternity leave to connect with friends and coworkers. Nonetheless, compared to an earlier online survey conducted in August 2013 (*n* = 1005) using the same questions, more women in our study perceived maternity leave as a benefit to mothers’ career (an increase of 19%), preferences (increase of 13%), and income (increase of 4%) [42], suggesting that the acceptance of longer paid maternity leave and its benefits for mothers and families is increasing in Vietnam. However, the fact that fewer women in our sample perceived that the policy was beneficial to employers (decrease of 3%) [42] indicates that many female workers continue to perceive maternity protection as a detriment to employers.

Female employees reported discrimination in hiring and employment practices based on pregnancy and childbirth including requiring proof of non-pregnancy status, disclosure of plans to have a baby or marital status, and even disciplinary action related to pregnancy and childbirth. Although Vietnam emphasizes the equal rights of male and female citizens in all fields and affords special protections to women and children [43], the Labor Code (2012) in force at the time of data collection did not explicitly prohibit sex-based discrimination [27]. The revised Labor Code (2019), which came into effect on 1 January 2020, addressed this gap by explicitly prohibiting employment discrimination based on factors, including gender and sex, pregnancy, marital status, and family responsibility, among others [29]. Effort is required to ensure that policy makers, employers, and employees are aware of this change and support its implementation and enforcement. Effort is required to ensure that policy makers, employers, and employees are aware of this change and support its implementation and enforcement.

Upon returning to work, one third of women in our sample reported not feeling fully recovered from childbirth, including those who took all six months paid maternity leave. This finding calls into question whether having the option to return to work after four months, with doctor’s approval [27,28,29], is beneficial for mothers’ mental and physical well-being. Furthermore, only 62% of respondents reported working fewer hours during pregnancy and after giving birth compared with before pregnancy. The remaining 34% report working the same hours and 4% reported working longer than before maternity leave. Coupled with the fact that a quarter of mothers reported not receiving their paid breastfeeding break, these findings call for the need to increase female employees’ awareness of their right to negotiate for additional unpaid leave and to improve monitoring of employer compliance with local regulations to promote breastfeeding and maternal well-being in the workplace.

### Study’s Strengths and Limitations

This study has several strengths, including the use of in-depth interviews and quantitative survey with the adaptation of ILO questionnaires [1]. Enumerators were thoroughly trained and used hand-held devices, which helped to reduce errors during data entry and collection, facilitate data checking at the central level, and expedite analysis. To our knowledge, few peer-reviewed papers have reported both qualitative and quantitative data related to maternity protection from female employees in diverse provinces in Vietnam.

We also acknowledge several limitations to our study. The cross-sectional design with descriptive data analysis can only provide a snapshot of the situation in purposively selected provinces of Vietnam. The study design limits the generalizability of the findings on the uptake of maternity protection policies in Vietnam or elsewhere. Without asking about the duration of contribution to the public social insurance fund during the 12 months prior to giving birth (eligible criterion to receive the entitlement), it is difficult to know the proportion of women who were eligible to receive cash support but did not receive them. Nonetheless, analysis of data from a sample population of women working in the formal sector and contributing to the public social insurance fund helps us to gain an overall picture of the perspectives and experience of maternity protection among formally employed women in Vietnam. In-person data collection occurred during the COVID-19 pandemic, which made some women hesitant to participate in the interview (non-response rate of 14.6% in the initial enquiry for the meeting). The pandemic may also have caused us to underestimate the proportion of women returning to work before completing their six months maternity leave, due to the reduced demand for workers in many sectors. It is also possible that some mothers chose not to return to work due to concerns related to COVID-19 (i.e., fear of infection at work).

## 5. Conclusions

Vietnam’s advanced maternity protection policies help protect the rights of women and children. In our study sample of formally employed women in diverse settings, there was high awareness and uptake of maternity entitlements, and respondents understood the value of these entitlements for the health and well-being of their families and communities. However, we identified some implementation gaps, including a lack of knowledge of the full set of entitlements provided by law, discrimination based on pregnancy and childbirth, and perceived financial and non-financial barriers to the utilization of their entitlements. The findings also suggest disparities in the awareness and uptake of maternity entitlements by occupation (blue- vs. white-collar jobs) and sector (e.g., state owned, foreign investment, vs. private employers) that must be addressed to ensure that all female workers can fully benefit from maternity protection.

The data collection was conducted at the beginning of the COVID-19 pandemic, and there were many changes in the following two years, such as the disruption of employment and contributions to social health insurance, availabilities and types of job opportunities, and migration of work forces. Future study would be needed to explore the impact of COVID-19 on labor force participation, gender equity, and the implementation of the labor code.

## Figures and Tables

**Table 1:** Socio-economic characteristics of formally employed women by job category ^1^.

	Overall (*n* = 494)	Blue-Collar (*n* = 268)	White-Collar (*n* = 226)
Kinh ethnicity	94.5	92.5	96.9
Married	99.8	100.0	99.6
Living with husbands/partners	96.8	97.0	96.5
Highest level of education obtained:			
Secondary school or less	47.4	78.7	10.2
Diploma	22.5	15.3	31.0
Bachelor’s degree or higher	30.2	6.0	58.8
Contributed to the public social insurance fund	91.7	90.7	92.9
Sample status:			
Pregnant women	21.7	18.3	25.7
Mothers of an infant	78.3	81.7	74.3

^1^ Data were presented in percentage.

## Data Availability

Requests for data may be directed to the corresponding author and are subject to institutional data use agreements.
